# BCO App: tools for generating BioCompute Objects from next-generation sequencing workflows and computations

**DOI:** 10.12688/f1000research.25902.1

**Published:** 2020-09-16

**Authors:** Nan Xiao, Soner Koc, David Roberson, Phillip Brooks, Manisha Ray, Dennis Dean

**Affiliations:** 1 Seven Bridges Genomics, Inc., Charlestown, MA, 02129, USA

**Keywords:** BCO App, BioCompute Object, Common Workflow Language, precisionFDA, Cancer Genomics Cloud, IEEE 2791-2020

## Abstract

The BioCompute Object (BCO) standard is an IEEE standard (IEEE 2791-2020) designed to facilitate the communication of next-generation sequencing data analysis with applications across academia, government agencies, and industry. For example, the Food and Drug Administration (FDA) supports the standard for regulatory submissions and includes the standard in their Data Standards Catalog for the submission of HTS data. We created the BCO App to facilitate BCO generation in a range of computational environments and, in part, to participate in the Advanced Track of the precisionFDA BioCompute Object App-a-thon. The application facilitates the generation of BCOs from both workflow metadata provided as plaintext and from workflow contents written in the Common Workflow Language. The application can also access and ingest task execution results from the Cancer Genomics Cloud (CGC), an NCI funded computational platform. Creating a BCO from a CGC task significantly reduces the time required to generate a BCO on the CGC by auto-populating workflow information fields from CGC workflow and task execution results. The BCO App supports exporting BCOs as JSON or PDF files and publishing BCOs to both the CGC platform and to GitHub repositories.

## Introduction

The BioCompute Object (BCO) is an IEEE standard (IEEE 2791-2020) titled *Bioinformatics Analyses Generated by High-Throughput Sequencing (HTS) to Facilitate Communication* ^1^. BCOs provide a systematic approach for documenting next-generation sequencing (NGS) data analysis workflows in order to facilitate communication of these complex computations between stakeholders ^2^. The need for the BCO standard emerged from the realization that documenting NGS data analysis tool choices and parameter settings is equally as crucial for ensuring reproducibility as documenting experimental methods ^3^. Whereas there are elaborate methodologies for documenting experiments, there is no gold standard for documenting NGS computation. Consequently, the goal of developing BCO software tools is to facilitate the generation and adoption of BCOs from a range of computational architectures in support of government, academic, and industrial applications.

The BCO, in its simplest form, supports the documentation of workflows through nine domains (provenance, usability, extension, description, execution, parametric, input/output, error, and top-level fields), each with two to twelve fields that specify domain characteristics (i.e., domain fields). The BCO supports documenting execution components (such as computational implementations and computational platforms) through the execution domain and the description domain. The specification aims to further clarify the workflow execution via the input/output domain and the error domain that defines expected errors. It also allows additional information describing the appropriate use of a workflow through the usability and parametric domains. A primary design principle of the standard is to reduce the effort required to create BCOs that conforms to the specification, by only requiring plaintext entries for each field. The simplest BCO instantiation, by definition, is a JSON file with text entries corresponding to the domain fields.

We present the BCO App, a web application that assists in the rapid generation of BCOs from bioinformatics workflows and their execution results. The application accepts plaintext user inputs, workflow contents written in the Common Workflow Language (CWL), and task execution results from the Cancer Genomics Cloud (CGC), an NCI funded computational platform ^4^ and other similar informatics platforms. By connecting to the CGC, the application enables the users to automatically populate the workflow metadata, the fields in the execution domain, the fields in the input/output domain, and the fields in the parameter domain, which already exist within workflow written in CWL and task information on the CGC. Reusing workflow and task information reduces the time required to construct a BCO and allows users to focus on authoring content for description domains and usability domains. The BCO App can be deployed and accessed on local machines, dedicated hosting servers, and the CGC. Additional details on the supported running environments and cloud platform integrations can be found in the “Deployment” section. The application’s implementation and operation details are described below. An example bioinformatics pipeline for RNA-seq differential expression analysis is used to demonstrate the BCO generation flow.

## Methods

### Implementation

**Figure 1.**
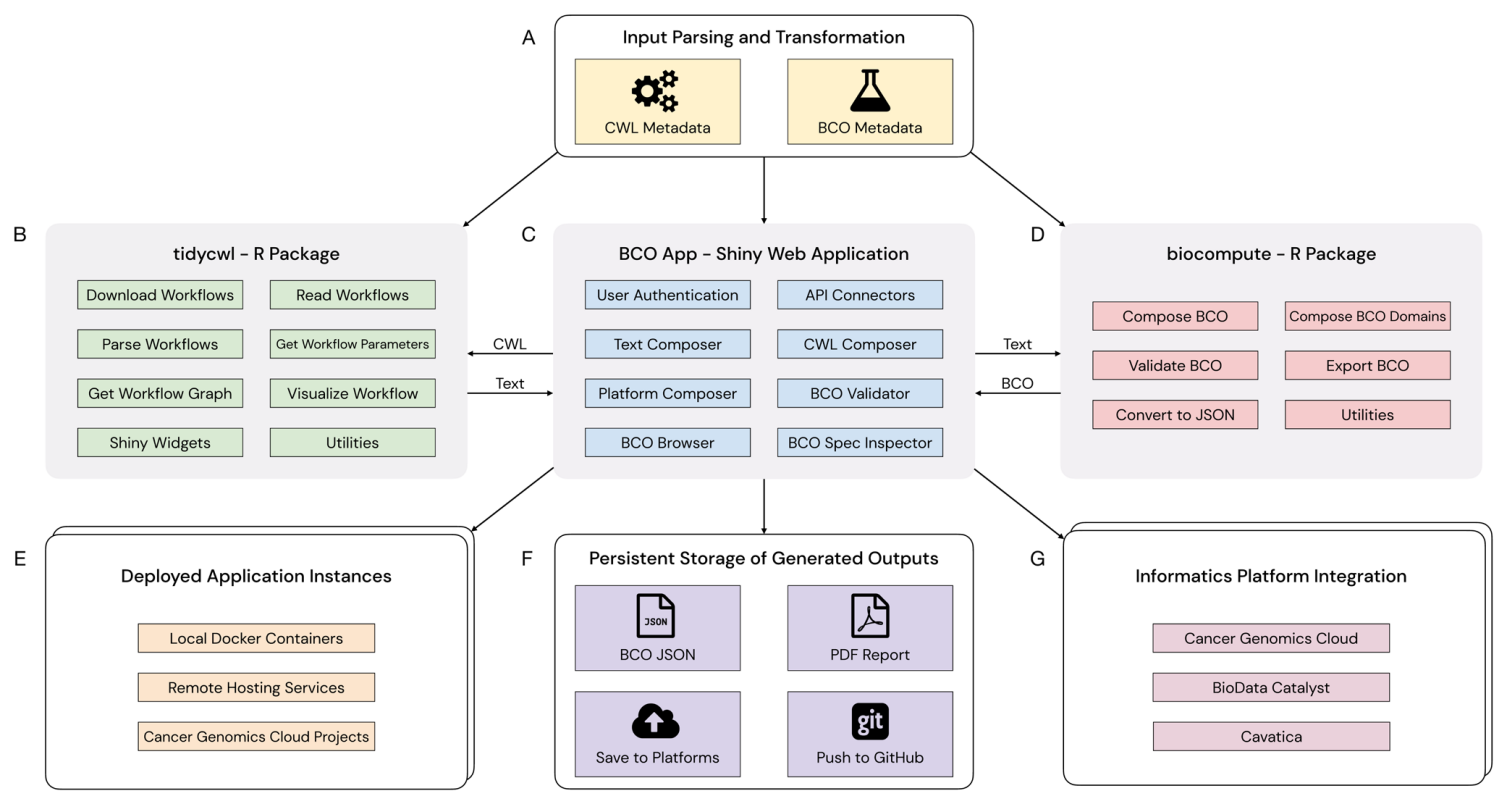
A schematic diagram of the BCO App’s architecture. **A**) The BCO App allows for text, workflows written in Common Workflow Language (CWL), and CGC task information as inputs. The BCO app parses complex inputs, as in the case for CWL workflow files and CGC task information. For the CWL input case, the BCO App extracts workflow metadata required to generate a BCO from the CWL file. **B**) The R package tidycwl handles CWL workflow processing, including reading, parsing, and visualizing CWL workflows. **C**) The BCO App offers BCO generation and validation capabilities powered by the tidycwl and biocompute R packages. **D**) The biocompute package implements the BioCompute standard. The package can compose, validate, and export BCOs. **E**) The BCO App can be deployed locally or remotely, with a fully-managed or self-managed approach. **F**) BCOs generated by the BCO App can be exported as JSON and PDF, or persistently saved to informatics platforms or GitHub repositories. **G**) The BCO App offers informatics platform integration that can be easily extended to include additional integration options.

Figure 1 shows an overall schematic of the BCO App’s architecture. The web interface is the central component of the application ( Figure 1C). The web interface provides an optional authentication module, accepts user inputs, supports interactive updates to the BCO field entries, displays generated outputs, and can optionally connect users to informatics platforms via an API. The backend of the web application ( Figure 1B, Figure 1D) receives user inputs, including workflow information ( Figure 1A), and composes the BCO output as either a JSON file or a PDF formatted file ( Figure 1F). The BCO App supports multiple deployment options, including local workstation support through a Docker container, persistently running instances on a remote hosting server, and the CGC ( Figure 1E). The modularized application also allows a user-contributed extension component to add support for additional cloud-based informatics platforms ( Figure 1G).

We use the R web framework Shiny ^5^ to implement the user interface and interaction logic of the BCO App. The functional components behind the application are two R packages: biocompute and tidycwl. The biocompute package is an implementation of the BioCompute standard in R. The package offers the capabilities to compose, validate, convert, and export BioCompute Objects. The tidycwl package can read, parse, and visualize CWL workflows from their JSON or YAML representations. These packages ensure that the application’s core components are separate from the interface code and interaction logic, while still being standardized and reusable for other applications developed for working with BCO and CWL. The architecture of both R packages employs the tidyverse design guide to ensure their consistency and interoperability within the existing R package ecosystem.

### Operation

In this section, we provide a summary of the BCO App’s features and deployment options. See the “BCO App User Manual” for more installation and operational details.

The BCO App architecture supports the generation of a BCO through the web application or by using the R packages biocompute and tidycwl directly. For advanced users or developers who prefer creating BCOs programmatically, please see the vignette “A Grammar for Tidying CWL Workflows” for processing CWL workflows, and the vignette “Create and Manipulate BioCompute Objects with R” for generating BCOs.

***Features.*** The primary features of the BCO App include 1) the BCO Composers, 2) the BCO Validator, and 3) the BCO Browser, with each feature arranged as an individual page accessible from the navigation bar. The application includes an optional authentication module, which allows the application administrators to control user access and manage permissions in scenarios such as collaborative BCO editing for a team of contributors and reviewers. Users can quickly search and browse definitions of specific BCO domains or fields from an interactive, tabular version of the BioCompute standard by visiting the “Utilities - Standard” page without losing the BCO content editing progress or focus. We describe the primary features below.

*BCO Composers*. The BCO App includes three types of composers that facilitate each of the three use cases driven by the source and type of inputs, detailed as follows:

- The Text Composer features a form wizard user interface for creating BCOs. This interface allows users to fill out the standard BCO fields as forms with plaintext input. After paging through the forms that facilitate user editing of fields by the BCO domain, the user can generate and review the BCO presented in JSON format. There is an option in the final step to download the BCO as a JSON file.
- The CWL Composer generates BCOs with the computational workflow information from uploaded CWL files. It offers semi-automated generators for creating BCOs from local workflows written in CWL. Generation of the BCO proceeds similarly to the Text Composer after the workflow is uploaded and parsed, with options to download the BCO as a JSON or PDF file.
- The Platform Composer can generate BCOs with the workflow and its execution information from computational platforms. It takes a user-specified workflow or task (a completed workflow execution archive) as input. It then uses this input to pre-populated workflow execution-related fields defined in the standard. It also includes additional options to publish the generated BCO to a CGC project or to GitHub repositories.

*BCO Validator*. The BCO Validator supports the two types of validation recommended by the BioCompute standard (IEEE 2791-2020). After uploading a BCO file, the validator computes and validates the SHA-256 checksum of all non-top-level domains, to ensure its content integrity. Next, the validator verifies each BCO domain against the BCO JSON schema and advises users about potential structural issues, such as a type mismatch or required fields being left blank.

*BCO Browser*. The BCO Browser includes an interactive BCO viewer that supports domain-specific BCO inspection, data type highlighting, collapsed/expanded view for nested BCO components, and copying the components selectively to the clipboard for further inspection.

***Deployment.*** The BCO App supports multiple testing and production scenarios by offering flexible, off-the-shelf installation or deployment options. Currently, there are three options to deploy and access the application.

*Self-managed local installation*. We offer a containerized version of the application, with all software dependencies packaged as a Docker image. Users can pull the pre-built Docker image from Docker Hub, or build the image locally, then run the Docker container to start the application.

*Fully-managed cloud deployment*. A pre-configured application is packaged with required dependencies, and it can execute inside the “Data Cruncher” environment on the CGC. This method enables CGC users to access and run the application inside a CGC-hosted RStudio Server instance, directly facilitating access to over 500 public CWL tools and workflows on the CGC.

*Self-managed cloud deployment*. Users can choose to host the BCO App with a dedicated hosting server using their existing cloud infrastructure. This approach provides a self-managed solution with secure, browser-based access to the application, suitable for large-scale distribution within organizations.

These deployment options aim to maximize the deployment flexibility while lowering the deployment barriers due to possible constraints in software access and security policies. The BCO App user manual provides detailed steps and additional information regarding the deployment.

## Use cases

We demonstrate the process of generating a BioCompute Object using the BCO App with an NGS data analysis workflow and its execution results available from the CGC. We specifically use an RNA-seq workflow with publicly available NGS data from a study of bi-ventricular heart failure (accession number GSE120852) ^6^. The workflow demonstrates a complete RNA-seq data analysis procedure, beginning from raw FASTQ files and ending with differential expression and pathway enrichment analysis results.

We used the Platform Composer in the BCO App to generate a BCO from a completed RNA-seq workflow execution. The Platform Composer guides the user from workflow selection through six steps resulting in a generated BCO. The first step involves selecting a specific workflow on the CGC. The application then populated multiple BCO fields across multiple domains automatically. More specifically, the application successfully captured the 102 input files, 187 output files, and four workflow steps with their associated input and output parameter lists. The application then populated the appropriate fields in the description and input/output domain with the captured information.

**Figure 2.**
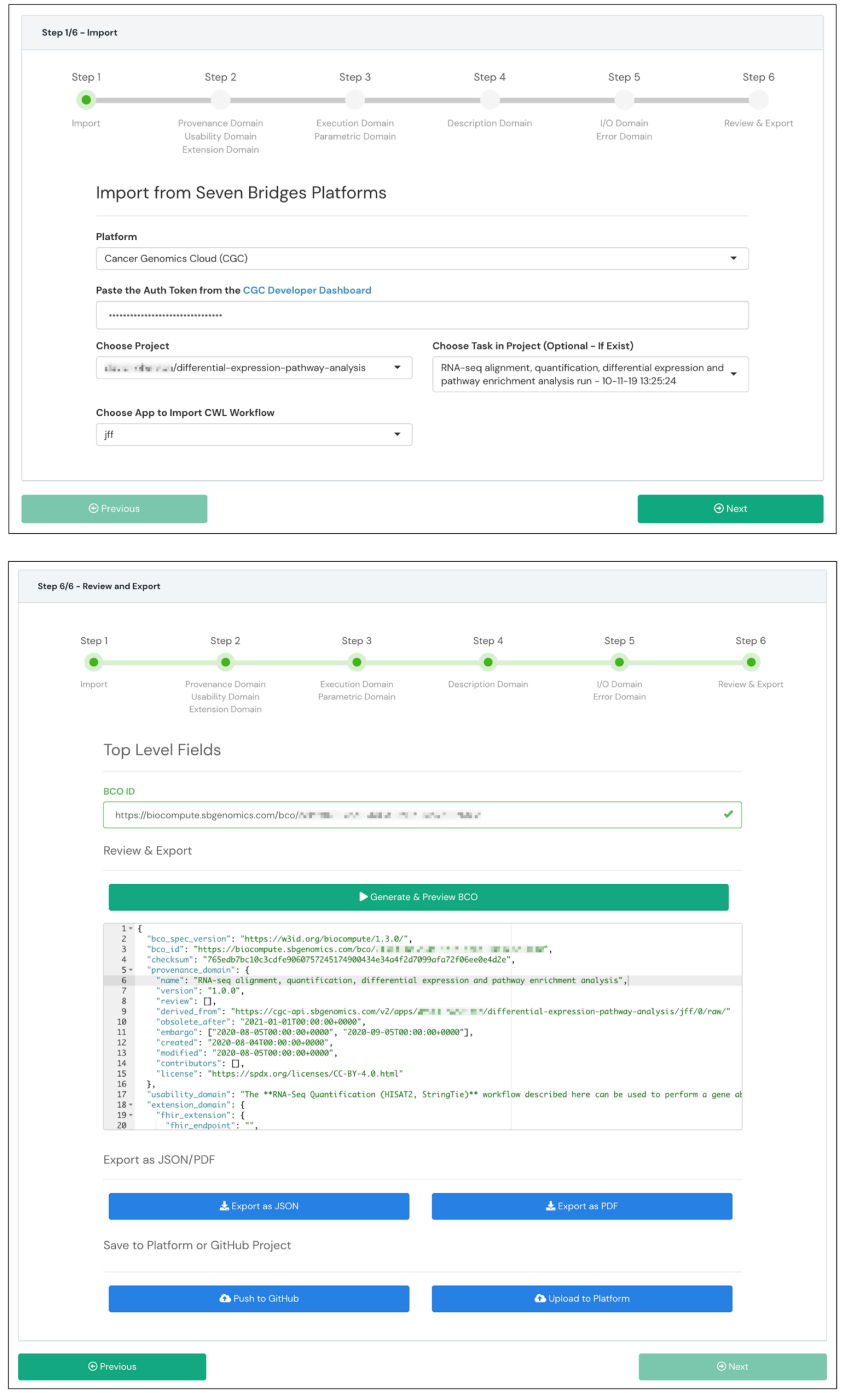
Selected forms from the Platform Composer generation wizard in the BCO App. The figure shows the first step (step 1) and the last step (step 6) for generating a BCO from a workflow stored on the Cancer Genomics Cloud (CGC). **A**) Step 1 shows the workflow import panel that includes the CGC project selector, workflow selector, task selector, and the authentication field. **B**) Step 6 shows the review and export panel that displays the BCO preview generated from the RNA-seq data analysis. The panel also shows buttons (features) to export the generated BCO as JSON or PDF and save it to the platform or GitHub repositories.

We then added additional workflow design details and a description of appropriate use to the usability domain. For the provenance domain, we provided detailed review and contributor information to ensure the traceability of changes made to the BCO. Finally, we exported the generated BCO as a JSON file. Figure 2 shows the first and the last form inputs (steps 1 and 6) of the BCO generation. See the *Data availability* section for additional screenshots taken during the BCO generation.

**Figure 3.**
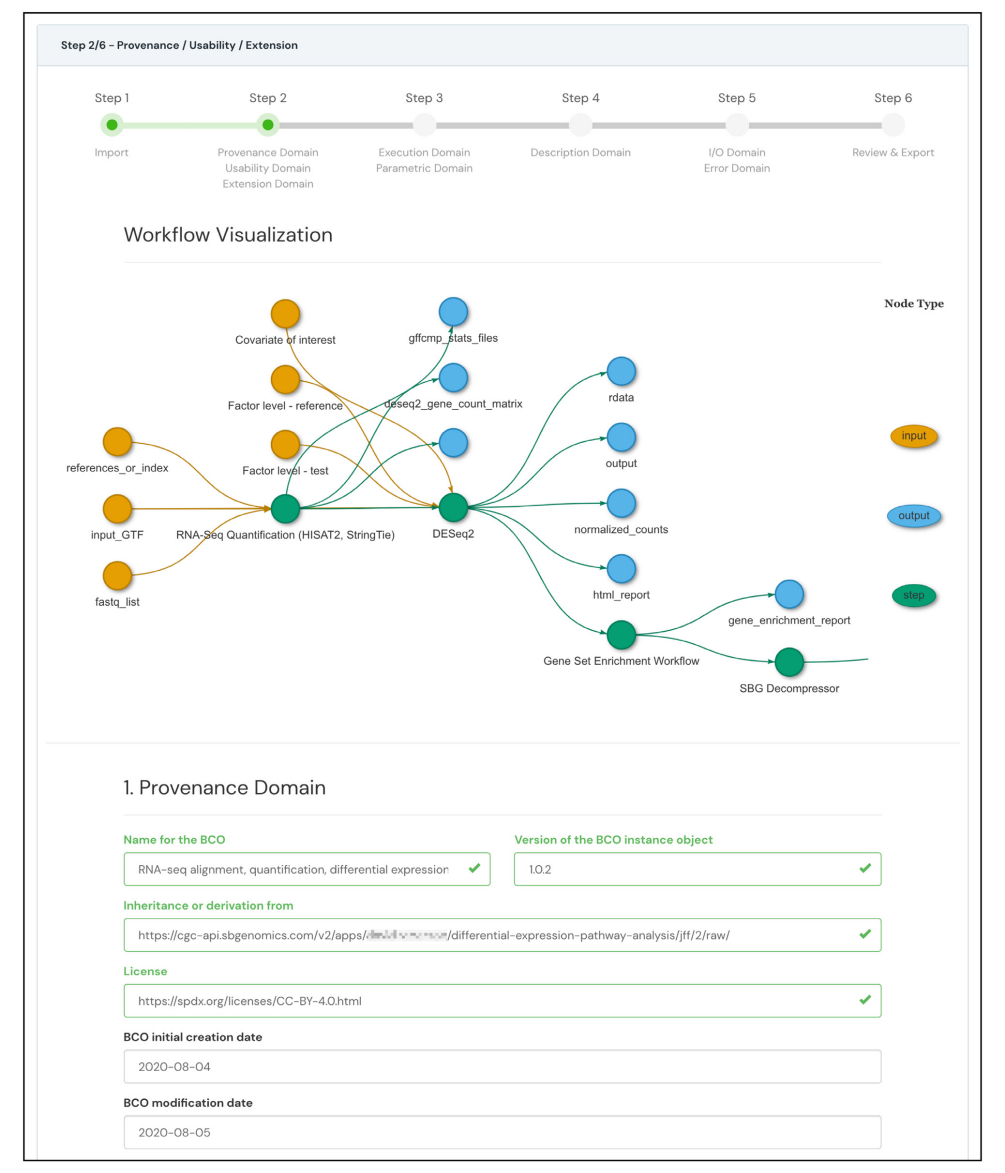
RNA-seq workflow wiring diagram constructed automatically by the information extracted from the CWL workflow. The figure highlights the automatically generated visualization of the RNA-seq workflow in the provenance, usability, and extension domain’s forms (step 2 of 6). The figure also shows some of the automatically populated fields in the provenance domain.

A major advantage of using CWL-based input is that the BCO App can access all the information within the CWL file, including the structured data that describes the workflow inputs, outputs, and steps. With the workflow graph data, the application can automatically generate a workflow wiring diagram which allows the user to review the workflow visually. Figure 3 presents the automatically constructed RNA-seq workflow visualization with the provenance, usability, and extension domain’s forms (step 2).

Notably, we submitted the generated BCO to the beginner track of the precisionFDA BioCompute Object App-a-thon in October 2019. The generated BCO received full scores on basic qualifications. The BCO App received high scores in terms of functionality, documentation, usability, and aesthetics as an advanced track submission.

The CWL workflow, the example RNA-seq data, and the generated BCO can be downloaded from the repositories mentioned under the *Data availability* section.

## Discussion

We developed the BCO App to facilitate the adoption of the BioCompute standard. Multiple practical use cases and deployment options are supported, from working on a local machine to working in cloud computing environments. Providing strong support for CWL processing makes documenting workflows more detailed, less error-prone, and reduces the time required to generate BCOs. Moreover, enabling the BCO App to access workflow and task information from the CGC exemplifies integrating the application with other informatics platforms. Designed with extensibility and modularity in mind, the application can be used as-is on platforms like the CGC. It can be easily extended to access workflow and task information from several other research platforms, including the NHLBI BioData Catalyst and Cavatica. Thus, BCOs and the BCO App could play a role in enhancing the computational reproducibility of NGS data analysis.

## Data availability

### Underlying data

The example BVHF RNA-seq dataset was obtained from NCBI GEO: WIPI1 is a Genetic Hub that Mediates Right Ventricular Failure, accession number GSE120852, and Sequence Read Archive (SRA), accession number SRP163468.

Figshare: BioCompute Object - RNA-Seq Differential Expression & Pathway Analysis - Generated by BCO App, https://doi.org/10.6084/m9.figshare.10257659.v4 ^7^.

This project contains the following underlying data:

- rnaseq-de-pathway.cwl.json (CWL workflow for RNA-seq differential expression and pathway analysis)
- rnaseq-de-pathway.bco.json (A BioCompute Object generated by the BCO App from the RNA-seq differential expression and pathway analysis workflow and execution results on CGC.)

### Extended data

Figshare: BCO App User Interface, https://doi.org/10.6084/m9.figshare.12793457.v2 ^8^.

This project contains the following extended data:

- 1-landing-page.png (Landing page)
- 2-composer-text-step-1...-5.png (Text Composer)
- 3-composer-cwl-step-1...-6.png (CWL Composer)
- 4-composer-platform-step-1...-6.png (Platform Composer)
- 5-browser.png (BCO Browser)
- 6-validator.png (BCO Validator)
- 7-standard.png (BCO Standard Viewer)
- 8-help.png (Application help page)

Data are available under the terms of the Creative Commons Zero "No rights reserved" data waiver (CC0 1.0 Public domain dedication).

## Software availability

Source code available from:

- https://github.com/sbg/bco-app (BCO App)
- https://github.com/sbg/biocompute (R package biocompute)
- https://github.com/sbg/tidycwl (R package tidycwl)

Archived source code at the time of publication:

- http://doi.org/10.5281/zenodo.3967760 (BCO App) ^9^
- http://doi.org/10.5281/zenodo.3967769 (R package biocompute) ^10^
- http://doi.org/10.5281/zenodo.3967767 (R package tidycwl) ^11^

License: GNU Affero GPL v3.

Access to the Cancer Genomics Cloud is free for all academic and nonprofit researchers, but it requires the creation of a login before use. Users can log in with either an email and password, or they can log in with their eRA Commons credentials to access controlled data. Data access restrictions according to each dataset apply. See here for more information: https://www.cancergenomicscloud.org/controlled-access-data.
