# Supplementary material for: BCO App: tools for generating BioCompute Objects from next-generation sequencing workflows and computations
Source: F1000Res. 2020 Sep 16;9:1144. [Version 1] doi: 10.12688/f1000research.25902.1 (PMC7702177; doi:10.12688/f1000research.25902.1)
Supplement: Peer review history

# Peer review: Reviewer response for version 1

**DOI:** 10.5256/f1000research.28585.r71509

**Published:** 2020-11-26

**Authors:** Yuriy Gusev

**Affiliations:** 1 Innovation Center for Biomedical Informatics (ICBI), Georgetown University Medical Center, Washington, DC, USA

A concept of biocompute objects (BCOs) was developed about 3 years ago to satisfy regulatory research needs for evaluation, validation, and verification of bioinformatics pipelines. Since then BioCompute Object (BCO) standard was established as an IEEE standard for communicating NGS data analysis pipelines primarilhy across regulatory agencies such as FDA. 

 This article describes a new web application that allows to generate BCOs on a range of computational platforms with major focus on cloud computational environments. Importantly, the BCO App accepts plaintext user inputs, workflow contents written in the Common Workflow Language (CWL), and task execution results from the Cancer Genomics Cloud (CGC), that has became one of the major platforms of choice for bioinformaticians conducting cancer genomics research. The BCO App provides fast and efficient way to generate BCO from existing CGC pipelines or workflows and task execution results. The BCO App can be extended to access workflow and task information from several similar cloud based computational platforms.

Are the conclusions about the tool and its performance adequately supported by the findings presented in the article?

Yes

Is the rationale for developing the new software tool clearly explained?

Yes

Is the description of the software tool technically sound?

Yes

Are sufficient details of the code, methods and analysis (if applicable) provided to allow replication of the software development and its use by others?

Yes

Is sufficient information provided to allow interpretation of the expected output datasets and any results generated using the tool?

Yes

Reviewer Expertise:

Bioinformatics, genomics data science, cancer research

I confirm that I have read this submission and believe that I have an appropriate level of expertise to confirm that it is of an acceptable scientific standard.

# Peer review: Reviewer response for version 1

**DOI:** 10.5256/f1000research.28585.r71511

**Published:** 2020-09-23

**Authors:** Ezekiel Maier

**Affiliations:** 1 Booz Allen Hamilton, McLean, VA, USA

This software tool article describes the BCO App, which enables users to easily generate of BioCompute Objects. BioCompute Objects are instances of the IEEE approved BioCompute specification, and provide a standardized reporting framework to enhance the reproducibility of computational workflows. The BCO App can be deployed locally or in the cloud (including the Cancer Genomics Cloud), and provides text, CWL, and platform based mechanisms for composing BCOs. In additions the BCO App provides a BCO validator to ensure compliance of the BCOs to the BioCompute specification, and a browser for viewing BCOs. The BCO App was designed and developed with modularity and interoperability in mind. The BCO App substantially reduces the burden of generating, validating, and viewing BCOs.

Are the conclusions about the tool and its performance adequately supported by the findings presented in the article?

Yes

Is the rationale for developing the new software tool clearly explained?

Yes

Is the description of the software tool technically sound?

Yes

Are sufficient details of the code, methods and analysis (if applicable) provided to allow replication of the software development and its use by others?

Yes

Is sufficient information provided to allow interpretation of the expected output datasets and any results generated using the tool?

Yes

Reviewer Expertise:

Bioinformatics

I confirm that I have read this submission and believe that I have an appropriate level of expertise to confirm that it is of an acceptable scientific standard.
